# Contribution of substantia nigra glutamate to prediction error signals in schizophrenia: a combined magnetic resonance spectroscopy/functional imaging study

**DOI:** 10.1038/npjschz.2014.1

**Published:** 2015-03-04

**Authors:** David M White, Nina V Kraguljac, Meredith A Reid, Adrienne C Lahti

**Affiliations:** 1 Department of Psychiatry and Behavioral Neurobiology, University of Alabama at Birmingham, Birmingham, AL, USA; 2 Department of Biomedical Engineering, University of Alabama at Birmingham, Birmingham, AL, USA; 3 Department of Electrical and Computer Engineering, Auburn University, Auburn, AL, USA

## Abstract

**Background:**

Because dopamine neurons signal a mismatch between expected and actual reward called prediction error (PE), aberrant PE signals in schizophrenia have been attributed to known dopaminergic abnormalities. However, dysfunction of *N*-methyl-D-aspartate receptors on cortical γ-aminobutyric acid neurons, as hypothesized in schizophrenia, could lead to excess glutamate release in the substantia nigra (SN) and affect reward processing.

**Aims:**

The aim of this study was to investigate the contribution of SN glutamate to PE signals in healthy controls (HC) and patients with schizophrenia (SZ).

**Methods:**

We recruited 22 medicated SZ and 19 HC. We obtained (1) functional magnetic resonance imaging during a probabilistic monetary reward task to assess PE-related blood oxygen level-dependent (BOLD) signal and (2) magnetic resonance spectroscopy to measure Glx (glutamate+glutamine) in the SN. To identify group differences in regions where the BOLD signal varies as a function of PE, we analyzed PEs generated during the task as parametric modulators of reward delivery. Finally, we examined the correlation of PE-related BOLD signal and SN Glx in each group.

**Results:**

Relative to HC, PE-related BOLD signals in SZ were significantly different in the midbrain/SN and ventral striatum. In SZ, SN Glx was significantly elevated. In HC, but not in SZ, PE-related BOLD signal in SN was positively correlated with SN Glx.

**Conclusions:**

These results suggest a role of glutamate in the neural coding of PE in controls. They indicate that glutamatergic dysfunction might contribute to abnormal PE coding in schizophrenia, suggesting the use of glutamate-targeted approaches to improve these deficits.

## Introduction

Reinforcement learning models posit that, to maximize reward, learning from our environment occurs by comparing expected outcomes with attained outcomes. Prediction error (PE) signals are generated when outcomes deviate from predictions, which leads to updating of reward expectations. Reward processing and, most specifically, PE are linked to dopaminergic function.^1^ Electrophysiological studies of midbrain dopamine neurons in primates show the firing of neurons increase when a reward exceeds what was predicted and decrease when a reward is less than predicted.^2^ In schizophrenia, it is hypothesized that known dopamine abnormalities^3^ could lead to aberrant encoding of PE signals.^4^ In this context, some symptoms could stem from aberrant attribution of salience to irrelevant stimuli, such as delusions, or from reduced attribution of salience to rewarding events, such as anhedonia.^5,6^ Imaging studies in schizophrenia have registered aberrant PE signals during reward processing and related these to symptoms.^7–10^

The revised glutamatergic hypothesis of schizophrenia proposes that blockade of *N*-methyl-D-aspartate receptors on γ-aminobutyric acid neurons could result in a disinhibition of glutamatergic neurons leading to excess glutamate release in projection areas.^11,12^ Because both the substantia nigra (SN) and the striatum receive glutamatergic projections from cortical areas,^13,14^ abnormal cortical glutamate transmission could affect these regions. Consistent with this model, a recent proton magnetic resonance spectroscopy (^1^H-MRS) study found higher glutamate+glutamine (Glx) levels in the striatum of antipsychotic-naive patients with schizophrenia.^15^ We previously reported the results of a ^1^H-MRS study of the SN in medicated patients. Although we did not observe differences in Glx, the Glx/*N*-acetyl-aspartate ratio was significantly elevated in patients, possibly indexing a glutamatergic dysfunction.^16^ Therefore, glutamate dysfunction in the SN could affect reward processing. However, little is known about the contribution of glutamate to reward both in general and also to its dysfunction in schizophrenia.

The aim of this study was to investigate the contribution of glutamate to PE signals in healthy controls (HC) and patients with schizophrenia. We combined functional MRI (fMRI) during PE processing with single-voxel ^1^H-MRS in the SN to obtain Glx measurements. We hypothesized that we would replicate findings of abnormal PE-related neural signals in the SN in patients. In addition, for the first time, we explore the contribution of Glx to PE-related neural signals and its implication in schizophrenia.

## Materials and methods

### Participants

We enrolled 22 medicated participants with schizophrenia or schizoaffective disorder (SZ), recruited from outpatient clinics at the University of Alabama at Birmingham, and 19 matched HC, recruited via advertisement. After being deemed able to give consent,^17^ informed consent was provided. Approval by the Institutional Review Board was obtained. All participants were recruited for a multimodal neuroimaging study of reward. Neurometabolite measurements of some participants have been included in another report.^16^

Diagnoses were established through review of medical records, two clinician consensus, and the Diagnostic Interview for Genetic Studies. Exclusion criteria were major medical/neurological conditions, pregnancy, substance abuse within 6 months of enrollment, and head injury with loss of consciousness. HC were without history of Axis I disorders or family history of psychosis. Cognitive functioning was characterized by the Repeatable Battery for the Assessment of Neuropsychological Status,^18^ and symptom severity by the Brief Psychiatric Rating Scale.^19^

### Reward task

**Figure 1:**
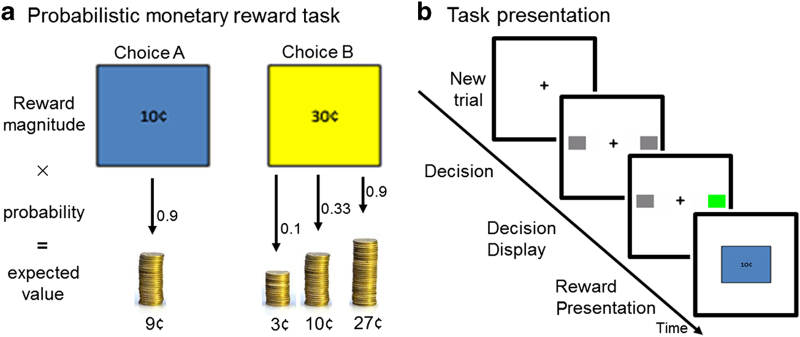
(**a**) Participants selected either a large reward of 30¢ or a smaller reward of 10¢ by pushing a right or left box. Although the probability of receiving 10¢ remained constant at 0.9, the probability of receiving 30¢ varied between runs (0.1, 0.33, or 0.9). After the first 10 trials of each run, participants developed an expected value (EV) (probability×reward magnitude (RM)) of their choice. Prediction error (PE) was calculated as the difference between RM and EV for each trial (that is, if EV=9¢ (0.9×10¢), but RM=0¢, then PE=−9). (**b**) Three conditions were presented. During Decision, subjects selected the left or right box corresponding to a 10¢ or 30¢ choice. For a given run, the left/right position of the 10¢/30¢ choice did not change. During Decision Display, the color of the box selected changed, indicating that a response was made. Feedback was received during Reward Presentation (RM of 0¢, 10¢, or 30¢).

After a training session, subjects performed a probabilistic monetary reward decision task modeled after Rolls *et al.* ^20^ during fMRI (six runs of 25 trials; Figure 1). Each trial consisted of three conditions (Decision, Decision Display and Reward Presentation). Each condition was displayed in a pseudo-randomly jittered fashion lasting 2, 4, or 6 s (total of 10 s per trial). During Decision, participants selected either a large reward of 30¢ or a smaller reward of 10¢ by pushing a right or left box. Although the probability of receiving 10¢ remained constant (0.9), the probability of receiving 30¢ varied between runs (0.1, 0.33, and 0.9). Participants were informed that the left/right position of the different reward amounts and probability of receiving the reward of higher magnitude would change from run to run, but remain constant within a given run. That is, for a given run, the left/right position of the 10¢/30¢ choice would not change. During Decision Display, to indicate a response had been made, the color of the box selected changed. During Reward Presentation, the reward magnitude (RM) earned during a given trial (0¢, 10¢, or 30¢) was displayed. Subjects were instructed to sample both sides offered in each run to determine which selection was more advantageous, with the goal of maximizing the amount of money earned. As previously reported,^20^ subjects took less than 10 trials to adjust to change in probability and develop an expected value (EV) of that run. After the 10th trial of each run, the EV (RM×probability) for selecting 10¢ throughout the run was 9¢ and, based on increasing probability levels, the EVs for selecting 30¢ were 3¢, 10¢, or 27¢. Thus, after the 10th trial, the task was such that subjects generated PE. PE was calculated as the difference between the RM for each trial and EV for that run (RM−EV; that is, if EV=9¢ (0.9×10¢), but RM=0¢, then PE=−9). PE could take any one of the following values: −27, −10, −9, −3, 1, 3, 20, and 27.

### Image acquisition

All scanning was done on a 3 T Siemens Allegra head-only scanner (Siemens, Erlangen, Germany). A high-resolution structural scan was acquired for anatomic reference (magnetization-prepared rapid gradient echo (MPRAGE); TR/TE/inversion time=2,300/3.93/1,100 ms, flip angle=12°, 256×256 matrix, 1 mm isotropic voxels).

fMRI data were acquired using the gradient-recalled echo-planar imaging sequence (repetition time/echo time (TR/TE)=2,000/25 ms, flip angle=70°, field of view=192 mm, 6 mm slice thickness, 32 axial slices). An IFIS-SA system (MRI Devices, Corp., Waukesha, WI, USA) running E-Prime (version 1.2, Psychology Software Tools Inc., Sharpsburg, PA, USA) was used to control stimulus delivery and record responses.

**Figure 2:**
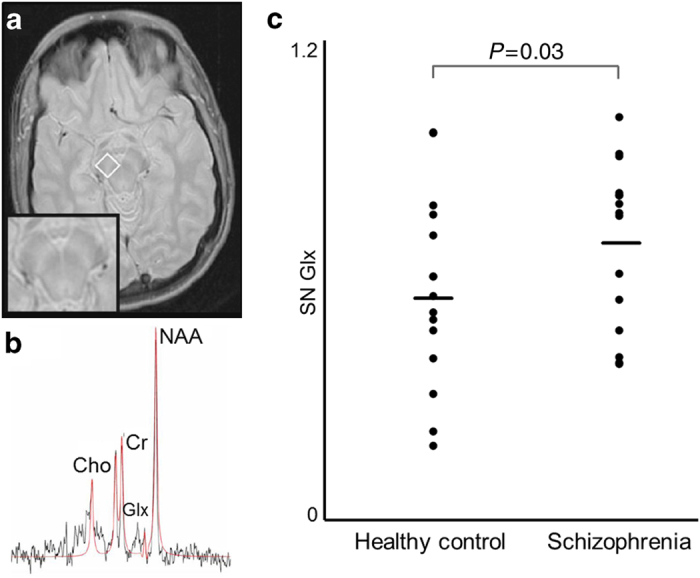
(**a**) Example of magnetic resonance spectroscopy (^1^H-MRS) voxel placement in the left substantia nigra (SN; 13×13×13 mm) overlaid on an axial magnetization transfer contrast image. Insert shows the midbrain without the ^1^H-MRS voxel. Images are displayed in neurological convention. (**b**) Sample ^1^H-MRS spectrum obtained from the left SN; the black line is a spectrum (640 averages), the red line is an overlay of the spectral fit. Cho, choline; Cr, creatine; Glx, glutamate+glutamine; NAA, N-acetyl-aspartate. (**c**) Glx in the left SN in healthy controls and patients with schizophrenia. Horizontal lines indicate group means.

We used a turbo spin echo sequence with magnetization transfer contrast to visualize the SN and aid in placement of an ^1^H-MRS voxel (13×13×13 mm; Figure 2) positioned around the left SN. Following manual shimming, water-suppressed spectra were collected with the point-resolved spectroscopy sequence (TR/TE=2,000/80 ms, 640 averages; for details see refs 16, 21).

### Statistical analyses

#### Demographics and behavioral data

Independent samples’ *t*-tests and *χ* ^2^-tests were used to compare groups on demographic and behavioral variables. A general linear model was used to determine whether HC and SZ performed the task in a similar manner. Each participant’s response during every trial was binarized to indicate a left or a right button press. These values were entered as the dependent variable in a linear regression. Fixed independent factors were entered to define each of the six sessions and each of the 25 trials. Group was entered as a random factor and participant identification was entered as a covariate. A planned contrast was conducted for the outcome of diagnostic status as a predictor of trial response.

### fMRI

Data were analyzed using SPM8 (Wellcome Trust, London, UK). Preprocessing included slice-timing correction, realignment, artifact and motion correction using ArtRepair, coregistration to the structural scan, normalization to Montreal Neurological Institute space, and smoothing (4 mm) using DARTEL.^22^

First-level analyses were conducted for each participant with a general linear model to determine the relationship between observed event-related blood oxygen level-dependent (BOLD) signal and regressors representing expected neural responses to trial events. To examine the effects of reward separate from learning, the first 10 trials of each run were excluded from analysis.^20^ Decision events (at the time of button press) and reward presentations (at the midpoint of the reward presentation window) were modeled as stick functions in the general linear model along with their first-order temporal derivatives. In addition, in order to identify regions where the BOLD signal changed as a function of PE, reward presentation events were parametrically modulated (correlated) by their respective PE, with values ranging from −27 to 27. The first-order PE regressor was orthogonalized to the reward presentation to ensure it was uniquely specified and validly estimated.^23^ Contrasts were carried forward to the second level for within- and between-group analyses. Whole-brain analyses were corrected for multiple comparisons using false discovery rate with significance level set to *P*<0.01. In addition, we conducted region of interest analyses using masks from the WFU pickatlas^24^ for the midbrain/SN (TD lobes) and bilateral ventral striatum/nucleus accumbens (IBASPM 71). The significance level was set to *P*<0.05 using small-volume corrections (SVC).

### ^1^H-MRS

^1^H-MRS data were quantified in the time domain, incorporating prior knowledge derived from *in vitro* and *in vivo* metabolite spectra (for details see refs 25–27). Cramer-Rao lower bounds, an estimate of uncertainty, were calculated for each peak; data with Cramer-Rao lower bounds >30% were excluded. Glx was quantified with respect to creatine, and will hereafter be referred to as Glx. Spectroscopy data were not obtained in 1 SZ completing the reward task, spectral quality was poor in 4 HC and 5 SZ, and 1 SZ was excluded as an outlier (>3 s.d. above mean), leaving 15 HC and 15 SZ in analyses involving Glx. An analysis of covariance with age and smoking as covariates was performed to assess group differences in Glx.

### Combined fMRI/MRS

Regression analyses were performed in SPM8 to identify regions in the midbrain/SN and ventral striatum where the linear relationship between PE and BOLD during Reward Presentation was correlated with SN Glx. The analysis was performed in HC and SZ using the same masks as above with SVC (*P*<0.05). To visualize the distribution of variance associated with these analyses, we extracted the first eigenvariate (from the main effect of PE-related BOLD signal for each individual) in the midbrain area where SN Glx was found to be correlated. We then plotted the first eigenvariate of the PE-related BOLD signal against SN Glx.

## Results

We found no differences in demographics, but SZ more commonly were smokers and, as expected, had lower Repeatable Battery for the Assessment of Neuropsychological Status scores. There were no differences between the groups in the amount of reward earned or the amount of PE generated by their response pattern. In addition, the analysis of task performance indicated that the distribution of choices made (right versus left) was not statistically different between groups (Table 1). SN Glx was significantly higher in SZ (0.69±0.21) compared with HC (0.57±0.24; *F*=5.60; *P*=0.03).

### fMRI results

In HC (Supplementary Table 1, Supplementary Figure S1), we found positive changes in the BOLD signal as a function of PE (positive PE-related BOLD changes) in the orbitofrontal cortex, bilateral caudate, angular gyrus, and occipital cortex, as well as negative PE-related BOLD changes in frontal regions including the anterior cingulate cortex, inferior frontal gyrus, parietal cortices, insula, basal ganglia, and thalamus that are largely consistent with prior findings.^20,28^

### Between-group analyses

**Figure 3:**
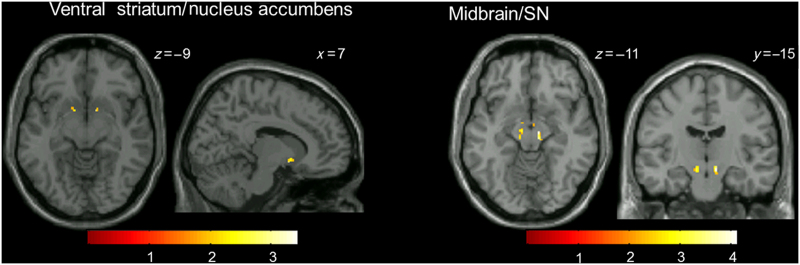
Areas where changes in BOLD signal as a function of PE (PE-related BOLD signal) were significantly different in patients with schizophrenia compared with healthy controls (analyses restricted to ventral striatum/nucleus accumbens (left) and midbrain/SN (right) using small-volume correction; *P*<0.05). Clusters are overlaid on a single-subject T1 structural image. The numbers adjacent to the slices indicate *y* and *z* coordinates in Montreal Neurological Institute convention for coronal and axial slices, respectively. The color bar indicates *t*-values. BOLD, blood oxygen level dependent; PE, prediction error; SN, substantia nigra.

PE-related BOLD signals in SZ (Supplementary Table 1, Supplementary Figure S1) were significantly different than those of HC in the following regions: inferior and middle frontal cortices, insula, caudate/ventral striatum, pallidum/putamen, thalamus, and midbrain/SN (also see Table 2). In region of interest analyses (Figure 3), PE-related BOLD signal was significantly different between SZ and HC in the ventral striatum/nucleus accumbens (cluster 1: *t*=3.45, *k*_E_=18, *x*=8, *y*=7, *z*=−7; cluster 2: *t*=3.05, *k*_E_=19, *x*=−16, *y*=9, *z*=−13) and the midbrain/SN (cluster 1: *t*=4.11, *k*_E_=1,414, *x*=6, *y*=−30, *z*=−12).

### Combined fMRI and MRS results

**Figure 4:**
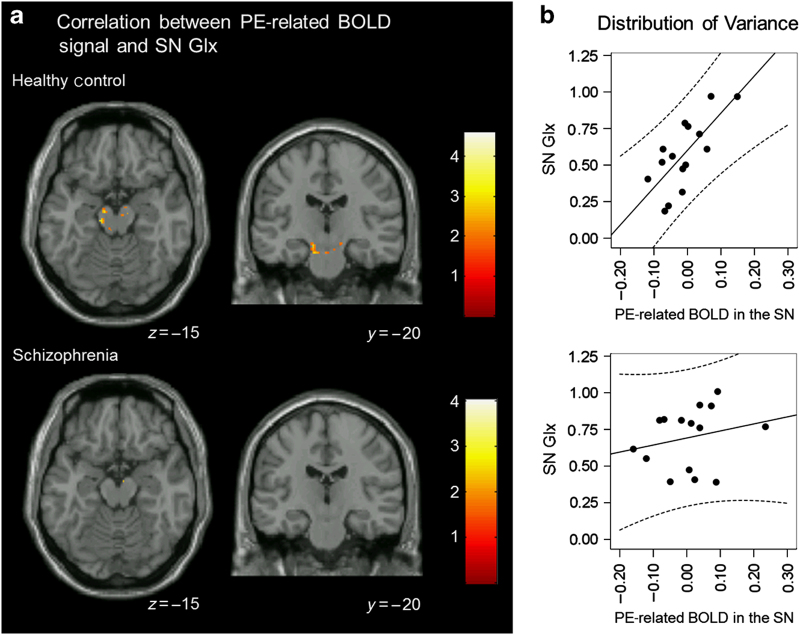
Correlation between PE-related BOLD signal and SN Glx. (**a**) In healthy controls, but not in patients with schizophrenia, there was a significant correlation between PE-related BOLD signal and SN Glx in the SN (analyses restricted to ventral striatum and midbrain/SN using small-volume correction; *P*<0.05). Clusters are overlaid on a single-subject T1 structural image. The numbers adjacent to the slices indicate *y* and *z* coordinates in Montreal Neurological Institute convention for coronal and axial slices, respectively. BOLD, blood oxygen level dependent; Glx, glutamate+glutamine; PE, prediction error; SN, substantia nigra. (**b**) Scatterplots showing the distribution of variance in the relationship between Glx and PE BOLD response in healthy controls (*r*=0.74) and patients with schizophrenia (*r*=0.30).

In HC, but not SZ, we found a significant correlation between the PE-related BOLD signal in SN and SN Glx (Figure 4a; *t*=4.60, *k*_E_=100, *x*=−12, *y*=−23, *z*=−19). Figure 4b scatterplots showing the distribution of variance in the relationship between Glx and PE BOLD response in HC (*r*=0.74) and SZ (*r*=0.30).

## Discussion

To our knowledge, this is the first study reporting the relationship between PE processing and SN Glx and its implications in schizophrenia. In SZ, we found abnormal PE-related neural response in the midbrain, ventral striatum, caudate, thalamus, orbitofrontal and dorsolateral prefrontal cortices as well as significantly elevated SN Glx. In HC, but not in SZ, the neural response to PE in the SN was positively correlated with SN Glx. These results suggest a role of glutamate in the neural coding of PE in HC, and that glutamatergic dysfunction might contribute to its abnormal coding in patients with schizophrenia.

Despite differences in experimental design and analyses, several studies in medicated and unmedicated patients have identified neural abnormalities during the encoding of PE, most prominently in the ventral striatum.^29^ Although some studies investigated reward-conditioning paradigms on a trial-by-trial level,^4,8,10^ others^7,9^ examined PE trials generated after conditioning completion like we did. Starting after the 10th trial, the behavioral analyses of our PE task show that SZ had learned the contingencies of the task during the first 10 trials. Compared with HC, there were no differences in the amount of reward earned or the amount of PE generated by their response pattern. In addition, the analysis of task performance indicated that the distribution of choices made (right versus left) were not statistically different between groups. This finding is consistent with the results of others.^30–32^ After the 10th trial, when the expected value of each trial was known to the participants, PEs were analyzed as parametric modulators of reward delivery. Like others,^4,7–10^ we found abnormalities of PE in dopamine-rich areas, including the SN, ventral striatum, caudate, and thalamus. In addition, in HC, correlation with the PE signal was also observed in dorsal anterior cingulate cortex and posterior parietal cortex, implying that executive processes were engaged. Interestingly, those regions were not differentially associated with the PE signal in patients, suggesting that abnormalities in PE in patients originate from bottom-up rather than top-down processing. Like others, we observed abnormal PE signals in patients who were medicated (mainly second-generation antipsychotics), indicating that dopamine D2 blockade does not normalize PE abnormalities. Interestingly, a study in first-episode patients found normalization of a reward anticipation-related ventral striatum hypofunction after treatment.^33^ It remains to be determined whether treatment does in fact reduce, but obviously not normalize, PE abnormalities, and whether treatment has a differential effect on PE dysfunction in first episode compared with chronic patients with schizophrenia.

Our finding of elevated SN Glx in SZ is consistent with previous finding of elevated Glx measured in the striatum,^15^ putatively suggesting excessive glutamate release from cortical glutamatergic projections to basal ganglia. However, although elevated striatum Glx was observed in unmedicated patients, our observations derive from medicated patients. The participants enrolled in this study overlap with those included in a prior report where, although no differences in Glx were observed, the Glx/*N*-acetyl-aspartate ratio was significantly elevated in patients, possibly indexing a glutamatergic dysfunction.^16^ Our findings are also consistent with the identification of abnormal expression of *N*-methyl-D-aspartate receptor-associated intracellular proteins in the SN in schizophrenia.^34^ In addition to cortical projections, glutamatergic projections to the ventral tegmental area originate from subcortical structures, including the subthalamic nucleus and the pedunculopontine tegmentum.^35^ Increased SN Glx might also reflect a local, rather than a projected disturbance. Recent optogenetic studies in rodents have demonstrated that mesolimbic dopaminergic neurons release glutamate in the nucleus accumbens, suggesting colocalization of glutamate and dopamine receptors in some midbrain neurons.^36,37^ Glutamatergic abnormalities have now been identified in schizophrenia in the basal ganglia, hippocampus, and medial prefrontal cortex.^15,38–40^ It remains to be determined whether these abnormalities originate from a similar local dysfunction, such as γ-aminobutyric acid interneuron abnormalities, or whether they are connected, one impacting the others, and the extent to which they are affected by treatment.

In HC, we observed a correlation between the PE signal and SN Glx in the SN. Given that the burst firing of dopamine neurons recorded during PE signals can be driven by application of glutamate to dopamine neurons or by stimulation of glutamatergic afferents,^41^ it is tempting to speculate that this correlation reflects the drive of glutamatergic projections to dopamine neurons in the SN. In the context of elevated SN Glx, the correlation between PE signal and Glx was not present in patients, suggesting that glutamatergic dysfunction could contribute to aberrant PE signaling. Consistent with our findings, low-dose administration of the *N*-methyl-D-aspartate antagonist ketamine disrupts error-dependent associative learning in healthy subjects.^42^

There are several limitations in our study. We quantified Glx using creatine as an internal reference because we did not collect unsuppressed water spectra or image an external phantom. As there may be creatine abnormalities in schizophrenia^43^ (but also see ref. 44), this study should be repeated using absolute quantitation. We did not correct Glx values for voxel gray matter content because of the limitations of our acquisition protocol. Future studies would benefit from acquiring a three-dimensional image with magnetization transfer contrast for the purposes of tissue segmentation.^45^ We used a large number of averages to increase the signal-to-noise ratio, which leads to a long scan time. Although this was well tolerated among our participants, it may not be ideal for all clinical populations. Our participants with schizophrenia were medicated, which could confound ^1^H-MRS measurements.^38,46^ Additional studies will be needed to determine the effect of antipsychotic medications. Illness stage and clinical status are important factors to consider in future studies, as they may also account for some variability in findings.

In summary, our results suggest a role of glutamate in the neural coding of PE and that glutamatergic dysfunction, such as the one we identified in the SN, might contribute to abnormal PE coding in schizophrenia. Because aberrant PE signals are found both in medicated and unmedicated patients, it suggests that dopamine D2 blockade may not reverse those deficits. Therefore, our results support the use of glutamate-targeted approaches to improve these deficits.

## Figures and Tables

**Table 1:** Demographics, clinical measures, and task performance^a^

	*SZ (*n*=22)*	*HC (*n*=19)*	t*/*X^*2*^	P*-value*
Gender (% male)	77.3	57.9	1.77	0.31
Age	39.41 (6.70)	36.47 (12.12)	−0.74	0.47
Parental occupation^b^	6.70 (5.05)	7.50 (4.76)	12.52	0.25
Smoking status (% smokers)	72.7	42.1	4.82	0.03
Smoking (packs per day)	0.66 (0.60)	0.36 (0.50)	−1.74	0.09
				
*Diagnosis*				
Schizophrenia	15			
Schizoaffective disorder	7			
Illness duration (in years)	17.68 (11.53)			
				
*Antipsychotic medication*				
First generation	2			
Second generation	17			
First and second generations	1			
Clozapine^c^	2			
				
*BPRS* d				
Total	30.27 (8.86)			
Positive	5.77 (3.74)			
Negative	4.27 (1.75)			
RBANS total index	76.14 (9.33)	93.32 (11.49)	5.28	<0.01
				
*Prediction error task*				
Total reward earned ($)	11.71 (1.59)	12.41 (1.14)	1.59	0.12
Mean prediction error ($)	−0.19 (0.33)	−0.31 (0.33)	−1.13	0.26
Task performance^e^	0.03 (0.01)	0.01 (0.02)	2.32^f^	0.13

Abbreviations: HC, healthy controls; RBANS, Repeatable Battery for the Assessment of Neuropsychological Status; SZ, schizophrenia.

a Mean (s.d.) unless indicated otherwise.

b Ranks determined from Diagnostic Interview for Genetic Studies (1–18 scale); higher rank (lower numerical value) corresponds to higher socioeconomic status. Parental occupation unknown in three HC and two patients with schizophrenia, *n*=36.

c One SZ with clozapine monotherapy and one SZ with combination of clozapine and ziprasidone.

d Brief Psychiatric Rating Scale (1–7 scale); positive (conceptual disorganization, hallucinatory behavior, and unusual thought content); negative (emotional withdrawal, motor retardation, and blunted affect).

e To assess diagnostic status as predictor of trial response, linear regression was conducted (button press as dependent variable, sessions and trials as fixed independent factors, and group as random factor). Values reported in parentheses are s.e.

f Reported value is F-statistic.

**Table 2:** Group differences in PE-related BOLD signal

*Brain regions*	*Voxels in cluster*	*Hem.*	*Voxels in region*	*Peak coordinates* a			*Peak* t
				X	Y	Z	
*PE-related BOLD signal*							
HC>SZ							
None							
SZ>HC							
Cluster 1	269			35	30	24	4.20
Inferior frontal cortex		B	156				
Middle frontal cortex		B	105				
Cluster 2	473			14	14	−2	2.87
Inferior frontal cortex		R	43				
Insula		R	23				
Caudate/ventral striatum		R	163				
Pallidum/putamen		R	108				
Cluster 3	346			18	11	15	4.81
Insula		R	10				
Caudate		R	168				
Putamen		R	22				
Cluster 4	540			6	−23	3	4.58
Thalamus		R	130				
Cluster 5	695			15	−36	−38	5.53
Midbrain/substantia nigra		B	695				

Abbreviations: B, bilateral; BOLD, blood oxygen level dependent; HC, healthy controls; Hem., hemisphere; PE, prediction error; R, right; SZ, schizophrenia.

a Reported in Montreal Neurologic Institute coordinates.
