# Supplementary material for: Contribution of substantia nigra glutamate to prediction error signals in schizophrenia: a combined magnetic resonance spectroscopy/functional imaging study
Source: NPJ Schizophr. 2015 Mar 4;1:14001–. doi: 10.1038/npjschz.2014.1 (PMC4752128; doi:10.1038/npjschz.2014.1)
Supplement: Supplementary Table S1 [file npjschz20141-s1.doc]

| **Table S1.** Prediction error related BOLD response in healthy controls | | | | | | | | |
| --- | --- | --- | --- | --- | --- | --- | --- | --- |
| Brain regions | Voxels in Cluster | Hem. | Voxels in Region | Peak Coordinates^1^ | | | Peak *t* |  |
|  |  |  |  | X | Y | Z |  |  |
| **Positive Prediction Error related BOLD** |  |  |  |  |  |  |  |  |
| Cluster 1 | 266 |  |  | 18 | 27 | 1.5 | 5.71 |  |
| Inferior orbitofrontal cortex |  | R | 3 |  |  |  |  |  |
| Caudate nucleus |  | R | 68 |  |  |  |  |  |
| Cluster 2 | 400 |  |  | -22 | -22 | 24 | 5.67 |  |
| Angular gyrus |  | L | 7 |  |  |  |  |  |
| Caudate nucleus |  | L | 6 |  |  |  |  |  |
| Cluster 3 | 409 |  |  | -21 | -93 | 3 | 7.10 |  |
| Fusiform/ lingual gyrus |  | L | 47 |  |  |  |  |  |
| Superior occipital cortex |  | L | 20 |  |  |  |  |  |
| Middle occipital cortex |  | L | 235 |  |  |  |  |  |
| Inferior occipital cortex |  | L | 102 |  |  |  |  |  |
| **Negative Prediction Error related BOLD** |  |  |  |  |  |  |  |  |
| Cluster 1 | 821 |  |  | -3 | -3 | 51 | 4.86 |  |
| Superior medial frontal cortex |  | B | 218 |  |  |  |  |  |
| Supplemental motor cortex |  | B | 443 |  |  |  |  |  |
| Middle cingulate cortex |  | B | 156 |  |  |  |  |  |
| Cluster 2 | 1747 |  |  | -34 | 12 | -3 | 5.86 |  |
| Superior frontal gyrus |  | R | 233 |  |  |  |  |  |
| Middle frontal gyrus |  | R | 44 |  |  |  |  |  |
| Inferior frontal gyrus |  | R | 646 |  |  |  |  |  |
| Precentral cortex |  | R | 210 |  |  |  |  |  |
| Cluster 3 | 962 |  |  | 35 | 12 | -3 | 5.10 |  |
| Inferior frontal gyrus |  | L | 233 |  |  |  |  |  |
| Rolandic operculum |  | L | 16 |  |  |  |  |  |
| Insula |  | L | 571 |  |  |  |  |  |
| Cluster 4 | 867 |  |  | 50 | 6 | -10 | 5.37 |  |
| Rolandic operculum |  | R | 114 |  |  |  |  |  |
| Insula |  | R | 214 |  |  |  |  |  |
| Pallidum/ Putamen |  | R | 47 |  |  |  |  |  |
| Cluster 5 | 642 |  |  | -21 | -66 | 54 | 3.94 |  |
| Superior parietal cortex |  | L | 262 |  |  |  |  |  |
| Inferior parietal cortex |  | L | 40 |  |  |  |  |  |
| Precuneus |  | L | 255 |  |  |  |  |  |
| Cluster 6 | 624 |  |  | -42 | -33 | 45 | 4.71 |  |
| Inferior parietal cortex |  | L | 452 |  |  |  |  |  |
| Cluster 7 | 889 |  |  | 36 | -63 | -37 | 4.95 |  |
| Inferior parietal cortex |  | R | 233 |  |  |  |  |  |
| Angular gyrus |  | R | 398 |  |  |  |  |  |
| Superior occipital cortex |  | R | 59 |  |  |  |  |  |
| Cluster 8 | 821 |  |  | -3 | -3 | 51 | 4.86 |  |
| Caudate |  | R | 156 |  |  |  |  |  |
| Putamen |  | R | 52 |  |  |  |  |  |
| Cluster 9 | 538 |  |  | -4 | -10 | -14 | 7.54 |  |
| Thalamus |  | L | 474 |  |  |  |  |  |
| Cluster 10 | 797 |  |  | 8 | -21 | -1 | 5.12 |  |
| Thalamus |  | R | 630 |  |  |  |  |  |
| Cluster 11 | 939 |  |  | 6 | -30 | -18 | 5.41 |  |
| Cerebellum |  | R | 158 |  |  |  |  |  |
| Abbreviations: Hem., hemisphere; L, left; R, right; B, bilateral ^1^Reported in MNI coordinates (X, Y, and Z). | | | | | | | | |
