# Supplementary figures and images for: Contribution of substantia nigra glutamate to prediction error signals in schizophrenia: a combined magnetic resonance spectroscopy/functional imaging study
Source: NPJ Schizophr. 2015 Mar 4;1:14001–. doi: 10.1038/npjschz.2014.1 (PMC4752128; doi:10.1038/npjschz.2014.1)

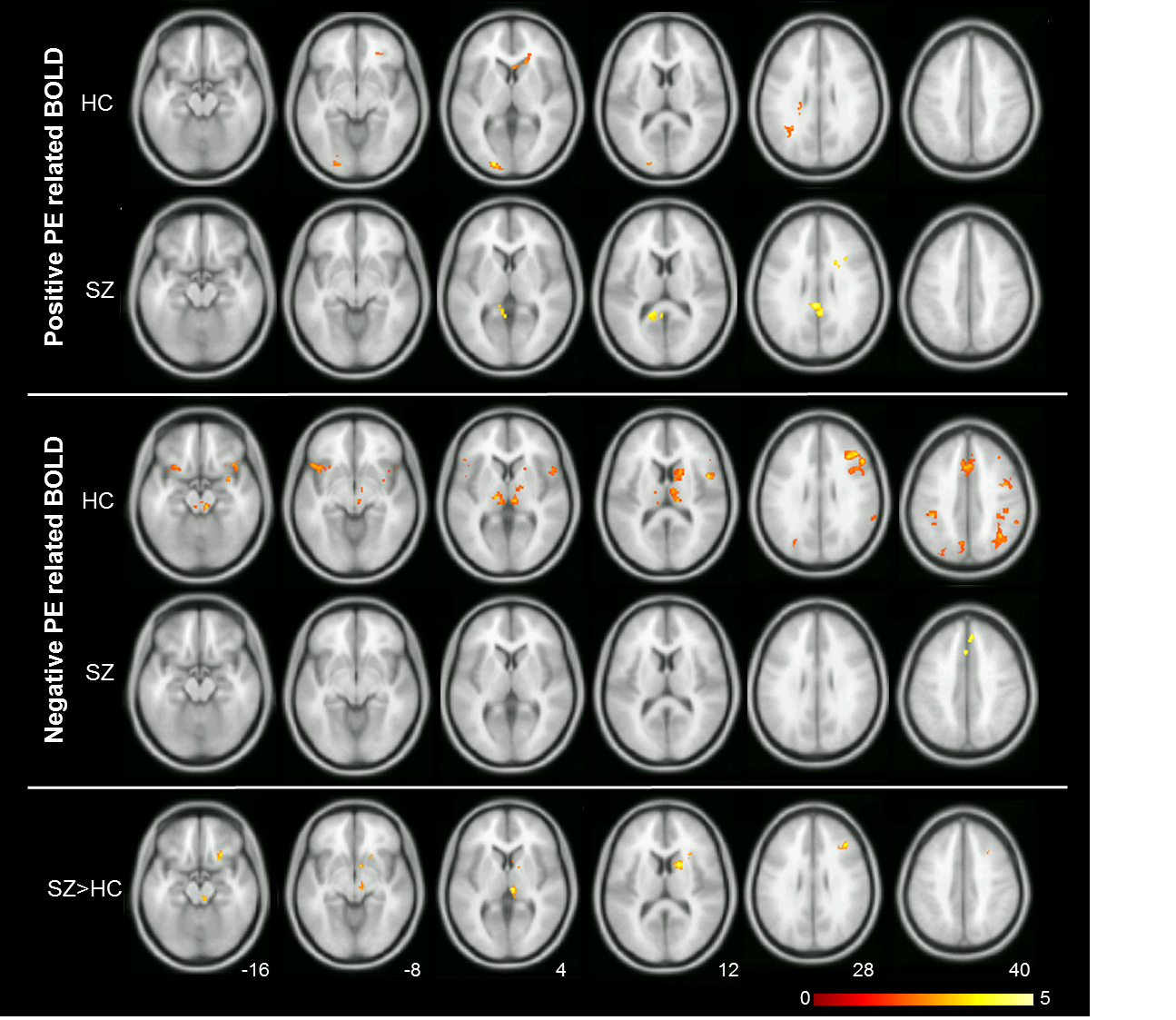

Supplement: Supplementary Figure [file npjschz20141-s2.tiff]
