# Supplementary material for: Contribution of substantia nigra glutamate to prediction error signals in schizophrenia: a combined magnetic resonance spectroscopy/functional imaging study
Source: NPJ Schizophr. 2015 Mar 4;1:14001–. doi: 10.1038/npjschz.2014.1 (PMC4752128; doi:10.1038/npjschz.2014.1)
Supplement: Supplementary Figure Legend [file npjschz20141-s3.doc]

**Supplementary Figure Legend:**

Top panel: Brain regions where positive changes in BOLD signal as a function of PE (positive PE-related BOLD signal) were significant in HC and SZ. Middle panel: Brain regions where negative PE-related BOLD signal were significant in HC and SZ. Bottom panel: Brain regions where changes in PE-related BOLD signal were significantly different between the groups. The numbers adjacent to the slices indicate z coordinates in MNI convention for axial slices. Whole-brain analyses were corrected for multiple comparisons using false discovery rate (FDR) with significance level set to *p*< 0.01. Clusters are overlaid on the avg152T1 brain template from the xjview toolbox. The color bar indicates t-values. BOLD, blood oxygen level dependent; HC, healthy control; PE, prediction error; SZ, schizophrenia.
